# LIFELONG CAREGIVERS: INFLUENCES OF AGE AND INCOME ON DEPRESSION AMONG PARENTS OF CHILDREN WITH DISABILITIES

**DOI:** 10.1093/geroni/igad104.2222

**Published:** 2023-12-21

**Authors:** Grace Udah, Carly Pullen, Julie Hicks Patrick

**Affiliations:** West Virginia University, Morgantown, West Virginia, United States; West Virginia University, Morgantown, West Virginia, United States; West Virginia University, Morgantown, West Virginia, United States

## Abstract

More than 20% of Americans have an intellectual or developmental disability (IDD). Of these, 70% are currently children and 30% are adults. Understanding wellbeing among their family caregivers provides an important avenue to supporting families. However, little research has included a wide range of family caregivers, focusing primarily of caregivers of young children or older caregivers of adults. Thus, it may be important to examine stressors among caregivers as they move across the lifespan. We used the 2020 BRFSS data to examine depression among a group of 522 family caregivers (M age = 56 years; range 24 – 80+) to children and grandchildren. Among the caregivers, 64% assisted with BADLs and 89% performed household chores related to the person with IDD. Among this age-diverse group, our 3-step hierarchical logistic regression revealed nonsignificant influences of caregiving tasks on depression, with Odds Ratios ranging from 1.11 to 1.22. Income was a significant contributor to the model, with ORs greater than 4.8 for the lower ends of income. After allowing caregiving tasks and income to account for their unique and shared variance, age still emerged as significant in Step 3, with younger caregivers somewhat less likely to report depression (OR = .98). Our results suggest the ongoing need to examine areas in which family caregivers need support across the lifespan.

